# Special feature on stem cells: current research and future prospects

**DOI:** 10.1038/emm.2013.131

**Published:** 2013-11-15

**Authors:** Il-Hoan Oh, Evan Y Snyder

**Affiliations:** 1 Catholic High-Performance Cell Therapy Center, The Catholic University of Korea, Seoul, Korea; 2 Sanford-Burnham Medical Research Institute, La Jolla, CA, USA

Stem cell-based therapies are viewed as among the most promising new strategies against developmental, traumatic, oncogenic and degenerative diseases. Indeed, it was the ability to identify, isolate, culture, interrogate, expand and transplant stem cells and/or their derivatives that gave rise to the new field of ‘regenerative medicine'. While the hope for stem cell-based regenerative medicine grows rapidly, it is becoming clear from extant clinical trials that we still need a greater understanding of the fundamentals of stem cell biology as well as of the disease process in order to make an impact that is efficient, efficacious, safe, reliable, scalable, cost-effective, practical, accessible and affordable.

During the past 20 years, the breadth of what is regarded as ‘stem cell research' has expanded immensely, now embracing, for example, tissue-specific, embryonic and induced/reprogrammed stem cells as well as cancer stem cells. Moreover, in the past 3–5 years, clinical trials using or relying on exogenous or endogenous stem cells (including cancer stem cells) have been launched with many more on the drawing board. With such rapid advances in the field, we felt it necessary to concisely take an overview of current thinking in stem cell biology, with a particular eye to potential applications.

In this special issue of *Experimental & Molecular Medicine*, we offer concise coverage of the state-of-the art in a series of representative areas in stem cell research provided by thought leaders in each topic.

First, the developmental changes in the characteristics of hematopoietic stem cells (HSCs), the one cobble stone model of tissue-specific stem cells, is discussed by Dr C Eaves. HSCs undergo extensive changes during development in their proliferation, self-renewal and growth factor responsiveness as well as their regenerative capacity. While the mechanisms underlying the shift in HSC properties remain largely unclear, this paper provides a recent view on the miRNA-mediated regulatory mechanisms that can define the developmental changes in HSCs.

Another type of stem cell, one that is sometimes shrouded in controversy, ‘very small embryonic-like stem cells (VSELs)', is discussed by Dr M Ratajczak. Dr Ratajczak addresses this controversy by describing their isolation, *in-vivo* identity and evidence for pluripotency based on transcriptomics and multi-lineage differentiation potential.

Dr E Snyder's group provides a recent view on the use of human-induced pluripotent stem cells (hiPSCs) for understanding the mechanisms underlying a complex disease (for example, bipolar disorder) based on responsiveness to a known clinically efficacious drug (for example, lithium). Based on a better understanding of lithium's heretofore unknown mechanism-of-action, then the same hiPSCs may be used to help screen and identify drugs that work better and with less toxicity than lithium against these same targets. In other words, in addition to being potentially useful for autologous cell therapy, hiPSCs can also serve as powerful tools for drug discovery based on their ability to be obtained from patients that harbor a disease and yield cells in a particular lineage that presumably also ‘have that disease'.

Moving to more applied stem cell biology, Dr A Caplan provides a concise review of mesenchymal stromal cell (MSC)-based therapy, describing basic concept as well as a mode of therapeutic action based on the secretion of factors that facilitate establishment of a regenerative microenvironment. Dr Caplan offers a spectrum of clinical studies ranging from immunological disorders to organ regeneration that have employed MSCs.

Dr J Yoo provides insight on the use of tissue-engineering scaffolds to facilitate a regenerative microenvironment in injured organs by stimulating the recruitment and proliferation of endogenous stem cells.

In addition, the current edition provides representative original researches covering each area of stem cell biology and therapeutic applications. The feasibility of using stem cell therapy in the treatment of neuronal diseases using neuronal stem cells and embryonic stem cell-derived neuronal progenitor cells as model systems is explored. Strategies for recruiting endogenous stem cells during wound healing and the possibility of salivary gland regeneration was explored. Finally, the clinical efficacy of stem cell therapy for femoral head necrosis was evaluated for 128 patients after 5 years of follow-up is discussed.

Collectively, these articles not only provide an overview of the state-of-the-art and future prospects for stem cell biology, but also describe the pipeline required to enable stem cell research to impact regenerative medicine.

We are particularly thankful to Dr Dae-Myung Jue, the Editor-in-Chief of *Experimental & Molecular Medicine* and to the Nature Publishing Group for their support on this special series ‘Special Feature on Stem Cells: Current Research and Future Prospects'.

